# Highly photoluminescent copper carbene complexes based on prompt rather than delayed fluorescence†
†Electronic supplementary information (ESI) available. CCDC 1469283 (for **1a**), 1469285 (for **1b**) and 1469284 (for **1c**). For ESI and crystallographic data in CIF or other electronic format see DOI: 10.1039/c6cc02349e



**DOI:** 10.1039/c6cc02349e

**Published:** 2016-04-18

**Authors:** Alexander S. Romanov, Dawei Di, Le Yang, Julio Fernandez-Cestau, Ciaran R. Becker, Charlotte E. James, Bonan Zhu, Mikko Linnolahti, Dan Credgington, Manfred Bochmann

**Affiliations:** a School of Chemistry , University of East Anglia , Earlham Road , Norwich , NR4 7TJ , UK . Email: m.bochmann@uea.ac.uk; b Department of Physics , Cavendish Laboratory , Cambridge University , Cambridge CB3 0HE , UK; c Department of Chemistry , University of Eastern Finland , Joensuu Campus , FI-80101 Joensuu , Finland

## Abstract

Linear two-coordinate copper complexes of cyclic (alkyl)(amino)carbenes (CAAC)CuX (X = halide) show photoluminescence with solid-state quantum yields of up to 96%; in contrast to previously reported Cu photoemitters the emission is independent of temperature over the range *T* = 4–300 K and occurs very efficiently by prompt rather than delayed fluorescence, with lifetimes in the sub-nanosecond range.

## Abstract 2

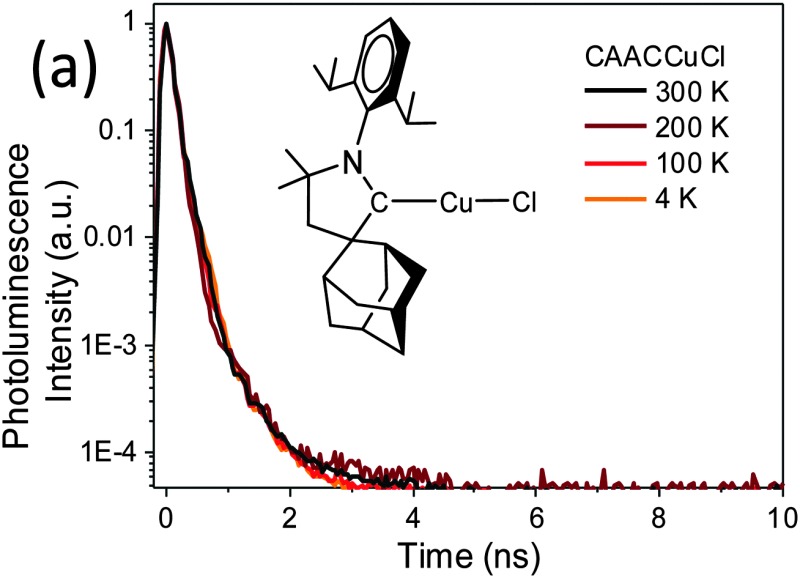
Simple carbene complexes of copper halides give photoluminescence quantum yields of up to 96%, with sub-nanosecond emission lifetimes.

Photoemissive complexes of copper(i) have been intensely investigated, not least in connection with possible applications as phosphors in organic light-emitting diodes (OLEDs).^1,2^ These are typically three- and four-coordinate complexes based on phosphine and heterocyclic ligands with good π-acceptor properties. Here, high coordination numbers and polynuclear halide-bridged structures have proved advantageous in minimising non-radiative decay, resulting in high quantum yields *via* the well-known “thermally activated delayed fluorescence” (TADF) emission pathway.^1–3^ More recently, three-coordinate copper complexes which oscillate between TADF and phosphorescence^4^ or show mixed emissions^5^ have also been described, with intrinsic exciton lifetimes of the order of 10–20 μs.^6^ For efficient OLEDs and phosphors, the emission lifetimes should be as short as possible to compete with non-radiative relaxation and to minimise self-quenching effects. Short lifetimes also allow for fast switching, enabling high optical data transfer rates.^7^

During recent studies on the oxidative addition reactions of gold(i) complexes stabilized by cyclic (alkyl)(amino)carbene (CAAC) ligands, we found that these compounds are unusually photosensitive.^8^ CAAC ligands are of course well-known for their strong σ-donor properties and have been used in numerous catalytic reactions^9^ but their photophysical properties have so far not been explored. We report here the synthesis, structure and photoluminescence properties of two-coordinate, monomeric CAAC copper and gold halide complexes. The copper compounds reach quantum yields of up to 96%.^10^ By contrast, analogous imidazolylidene-based NHC Cu and Au halide complexes are non-emissive.^11^ The ease of synthesis, good solubility in organic solvents, high thermal stability and non-self-quenching characteristics makes these complexes very promising as display phosphors and for incorporation into OLEDs. The complexes were prepared according to eqn (1).
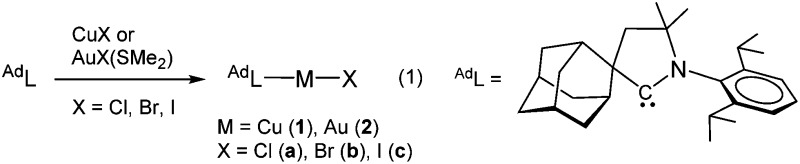


The compounds (^Ad^L)MX (M = Cu, Au; X = Cl, Br, I) are obtained in high yields. They show good solubility in polar non-protic solvents (dichloromethane, THF), are moderately soluble in toluene and insoluble in hexane. They are thermally very stable and can be sublimed at 200 °C/0.3 mmHg without decomposition. The thermal stability of the Cu and Au complexes as powders was determined by thermal gravimetric analysis (TGA) and differential scanning calorimetry (DSC) (for decomposition temperatures *T*_d_ and melting points *T*_m_ see ESI,† Table S1). The compounds melt above 270 °C, *e.g.* (^Ad^L)CuCl melts at 280 °C and decomposes above 328 °C.

Crystals of the copper halide complexes **1a–c** suitable for X-ray diffraction were obtained by layering of CH_2_Cl_2_ solutions with hexanes. The Cu complexes are two-coordinate and monomeric and crystallize with two independent molecules in the unit cell.‡
‡For the structures of the gold complexes **2** see ref. 8. The deviation of the C–Cu–X moiety from linearity decreases in the sequence Cl > Br > I, with angles of 175.33(5), 177.59(11) and 178.52(5)°, respectively (see ESI,† for details). The complexes show weak intermolecular C–H···Hal interactions.

Although CAACs are based on a saturated hydrocarbon skeleton, upon UV irradiation the free carbene shows blue fluorescence in the solid state, with a major emission band at *λ*_em_ = 447 nm (lifetime *τ* = 38.2 ± 0.1 ns), with low photoluminescence quantum yield (PLQY), *Φ*_PL_ = 9.1%; there is also a very weak emission at 660 nm (see ESI,† Fig. S1). Based on density functional theory (DFT) and time-dependent density functional theory (TD-DFT) calculations we assign these emissions to S_1_ → S_0_ and T_1_ → S_0_ transitions (*λ*calcem = 413 and 667 nm, respectively, allowing for geometric relaxation of the excited state).

The UV/vis absorption spectra of the copper and gold halide complexes in THF solutions show absorptions below 300 nm due to an intra-ligand transition of the CAAC ligand, as well as broad bands at 317 to 365 nm (see ESI,† Fig. S2 and S3). As DFT calculations show, the HOMO of the halide complexes changes from metal-halide σ for X = Cl to metal-halide σ* (for X = I) (Fig. 1; see also ESI,† Fig. S4). The low energy bands are assigned to a (σ + X)–π* charge transfer process,^12^ with only minor contribution from the metal. The photoexcitation in these complexes differs therefore from that observed in many copper halide complexes with phosphinopyridine-type ligands, which show predominantly metal–ligand charge transfer.^1,2,4,5^

**Fig. 1:**
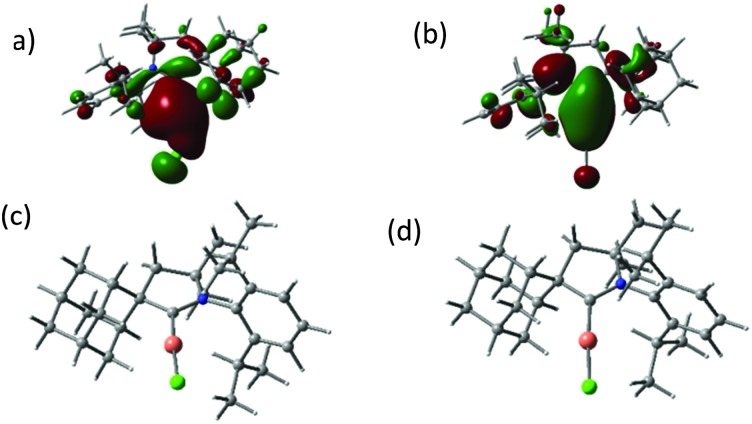
HOMO (a), LUMO (b) and geometry-optimised excited state structures T_1_ (c) and S_1_ (d) of (^Ad^L)CuCl (**1a**).

The redox potentials of **1** and **2** as determined by cyclic voltammetry, show one irreversible (X = Cl, Br) or partially reversible (X = I) reduction process which is centred on the metal atom. The reduction process becomes reversible in THF, as was previously noted for (^Cy^CAAC)AuCl.^13^ Oxidation of **1** and **2** exhibit one irreversible oxidation wave. The onset oxidation potential for gold halides shows more pronounced cathodic shift in the range Cl < Br < I. This suggests a greater contribution by the halide orbitals to the HOMO's energy level (see ESI,† Table S4).

The crystalline complexes display strong photoluminescence on excitation with UV light (*λ*_ex_ = 365 nm) (Table 1). Complex **1a** reaches a quantum yield of 96%; this compound shows a remarkably small geometric distortion in the excited state (Fig. 1c and d) which may contribute to the high emission efficiency. The complexes (^Ad^L)CuX appear to be the first examples of highly luminescent linear copper halides and show a bluish-white emission which is independent of X = Cl, Br or I. In contrast, the photoluminescence of the gold halides **2a–2c** shows a ligand-dependent red-shift, in the order X = Cl (blue) < Br (yellow-white) < I (yellow) (Fig. 2).

**Table 1:** Photophysical properties of (^Ad^L)MX complexes

Complex	*λ*_abs_ [nm] (10^3^ *ε*/M^–1^ cm^–1^), THF	Solid state	
		*λ*_em_ (*λ*_exc_) [nm]	*Φ*_PL_ ^*a*^
**1a**	290 (2.2), 365 (0.2)	453 (300–420)	0.96
**1b**	291 (7.8), 357 (0.4)	454 (300–420)	0.61
**1c**	298 (5.8), 352 (0.4)	460 (300–420)	0.28
**2a**	251 (7.6), 269 (sh, 4.1), 317 (0.2)	412 (280–375)	0.09
**2b**	257 (6.6), 284 (3.6), 316 (0.7)	480 (280–375)	0.13
**2c**	272 (5.1), 315 (4.2)	516 (280–375)	0.18

^*a*^Quantum yields determined by using an integrating sphere.

**Fig. 2:**
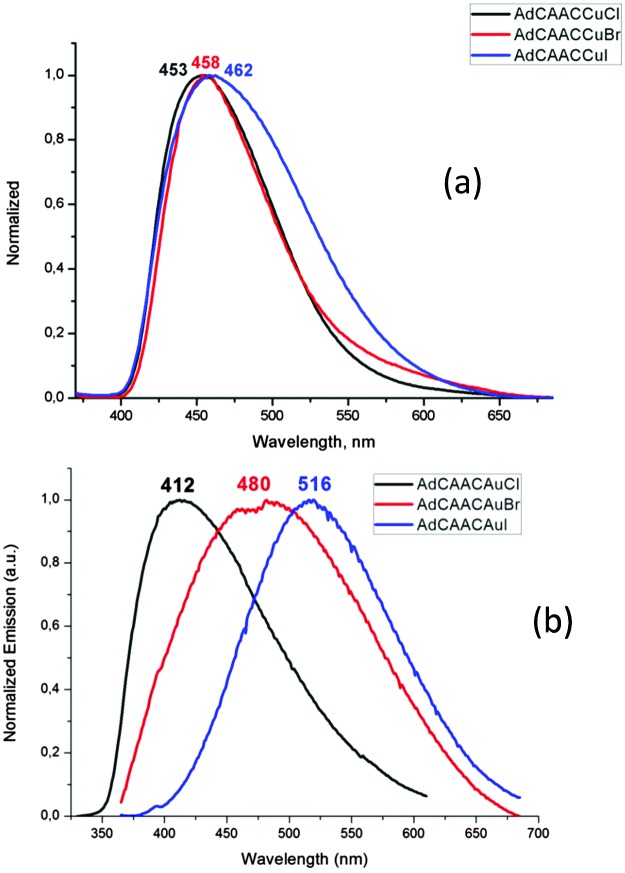
Emission spectra of (a) (^Ad^L)CuX and (b) (^Ad^L)AuX (X = Cl, Br, I) in the solid state (excited at *λ*_ex_ = 365 nm).

Photoemission by a TADF mechanism is characterised by a temperature-dependent blue shift, due to triplet-to-singlet upconversion, Δ*E*(S_1_ – T_1_) > 0.^1–5^ This process requires that the energy difference between the S_1_ and T_1_ excited states is sufficiently small, Δ*E* ≈ *k*_B_ *T*, to allow effective thermal population of both the T_1_ and S_1_ states. Since decay from S_1_ is much faster than from T_1_, the emission process is predominantly channelled *via* S_1_, with the delayed emission rate limited by the rate of inter-system crossing (ISC). Copper photoemitters have become synonymous with the TADF process. However, while this process provides an effective light harvesting mechanism from emitters for which radiative decay from the triplet state is forbidden, it is typically achieved by reducing the overlap between electron and hole orbitals, leading to low oscillator strengths and low radiative decay.^14^

Given the ample precedent for the TADF decay mechanism in copper complexes, it was surprising to find that the photoemissions of **1a–c** over the range of 4–300 K proved to be independent of temperature (see ESI,† Fig. S7). The normalised PL decay curves (Fig. 3a) for **1a** at different temperatures, as determined by time-correlated single photon counting (TCSPC), show that the emission is due to prompt fluorescence over 3 orders of magnitude, with a temperature-independent lifetime of only 0.22–0.3 ns (see ESI,† Fig. S8). This is close to the instrument response function width of 0.2 ns. The corresponding fluorescence decay rate is therefore at least 3.3–5 × 10^9^ s^–1^ for **1a**. There was no evidence for either delayed fluorescence or phosphorescence. As far as we are aware this is by far the highest fluorescence rate for an efficient copper-based photoemitter. Considering PLQY = *k*_r_/(*k*_r_ + *k*_nr_), where *k*_r_ is the fluorescence rate constant and *k*_nr_ is the rate of the non-radiative process incorporating both non-radiative singlet decay and ISC, we estimate a lower bound of 2 × 10^8^ s^–1^ for *k*_nr_ in **1a**.

**Fig. 3:**
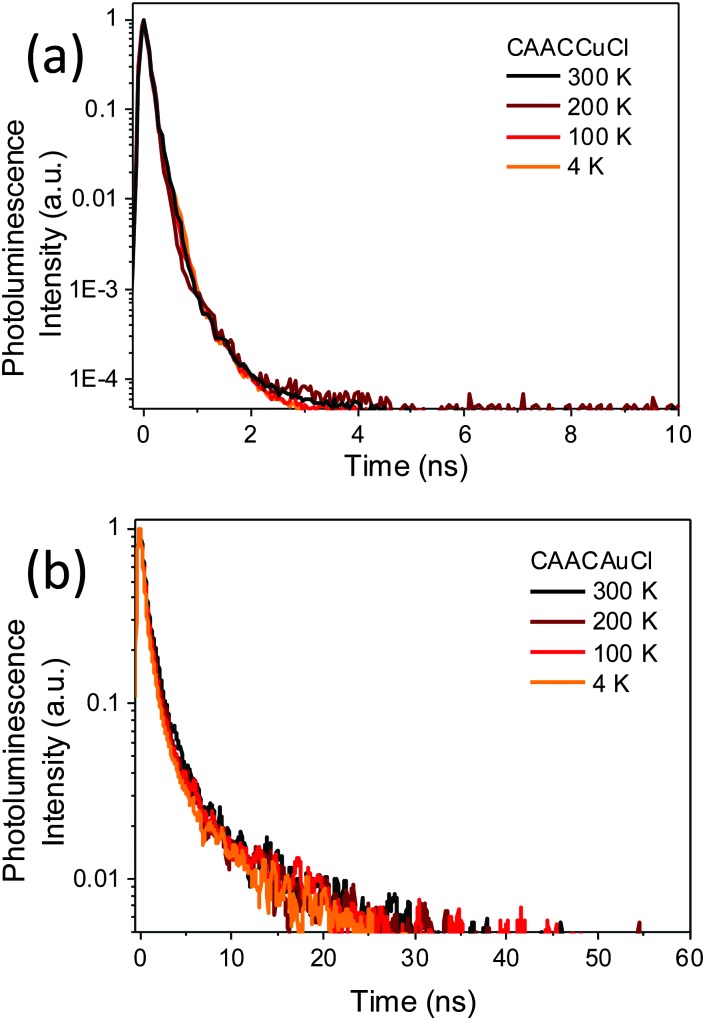
Normalised PL decay showing singlet prompt decay at different temperatures for (a) **1a**, and (b) **2a**.

Emission from the gold complexes **2** is also predominantly *via* temperature-independent prompt emission, though with substantially weaker PL, with lifetime of ∼0.5 ns (as shown for **2a** in Fig. 3b). A fluorescence rate of about 2 × 10^8^ s^–1^ can be estimated from its PLQY (0.09), much slower than the fluorescence rate of its Cu counterpart. The competing non-radiative decay rate is approximately 2 × 10^9^ s^–1^. There was no significant change in PL lifetime on cooling (see ESI,† Fig. S8) and, despite the presence of Au, there is no clear evidence for phosphorescence from this emitter. Since coinage metal complexes tend to exhibit strong spin–orbit coupling, sufficient to allow ISC on sub-ps timescales,^15^ the high fluorescence yield, lack of phosphorescence and magnitude of *k*_nr_ are surprising.

The unusual photophysical behavior was investigated with the aid of DFT and TD DFT calculations. The results reproduce the observed halide dependence of the excited state levels for the gold compounds and the lack of such ligand dependence in the case of copper. A comparison of the ground state and excited state geometries of **1a–1c** and **2a–2c** is given in Table 2 (see also ESI,† Fig. S5). The excited state structure of the copper chloride **1a** shows a remarkably small deviation from linearity (Fig. 1c and d); in fact the calculated C(carbene)–Cu–Cl angles in the T_1_ and S_1_ states (175.8 and 176.2°, respectively, see ESI,† Table S2) are essentially identical to the experimentally determined angle in the crystal (175.33(5)°). This aspect may contribute to the very high solid-state quantum yield. The excited states of **1b** and **1c** show more deviation from linearity, though not altering the identity of the LUMO (ESI,† Fig. S6), which leads to similar emission energies (Fig. 2a). The S_1_ – T_1_ energy difference of 30–40 kJ mol^–1^ is at the high end of values where a TADF process can be expected to operate, in agreement with the observation of prompt rather than delayed fluorescence from the S_1_ state in these copper halide complexes. Whereas the Au analogues show only slightly smaller S_1_ – T_1_ energy differences, the C–M–X angles in the S_1_ and T_1_ states are much more acute (ESI,† Table S2), in line with the lower radiative decay rate of **2a–c**. Spin mixing is most effective when singlet and triplet excited states exist which are near-degenerate.^15^ However, T_2_ energies are calculated to be about 0.3 eV above S_1_ for all compounds, consistent with slower ISC.

**Table 2:** Excitation energies (kJ mol^–1^) of halide complexes **1** and **2** in geometries optimized for the singlet and triplet excited states

Complex	T_1_	S_1_	S_1_ – T_1_
**1a**	248.0	285.5	37.5
**1b**	249.0	288.4	39.4
**1c**	249.3	285.4	36.1
**2a**	288.6	327.0	38.4
**2b**	281.9	318.4	36.5
**2c**	270.5	300.0	29.4

In coordinating solvents like THF the photoemissions of the copper halides **1a–c** show a solvent-induced red-shift of >130 nm. DFT calculations confirmed that while there is no THF coordination to the ground states of **1a**, **b** and **2a–c**, the binding energies to the T_1_ states are significant and are stronger for copper than for gold (see ESI,† Table S3).§
§Complex **1c** is an exception and makes a weak 3-coordinate THF complex in the ground state, Δ*E* = –9.2 kJ mol^–1^. The solvent-induced red-shift was therefore traced to emission from a three-coordinate solvent exciplex.

In conclusion, cyclic (alkyl)(amino)carbenes were found to be unexpectedly photoactive and give rise to linear, monomeric copper complexes which display photoluminescence quantum yields of up to 96%. Unlike three- and four-coordinate photoemissive copper complexes, for which high quantum yields have been correlated with temperature-dependent delayed fluorescence and which give decay lifetimes of several microseconds, the complexes reported here show temperature-independent prompt fluorescence, with decay times that are three to four orders of magnitude faster. This is sufficient to avoid ISC. Since triplet states are not involved, there is no requirement for dispersion to avoid self-quenching.^16^ The complexes are thermally very stable and sufficiently soluble and volatile for use in the fabrication of light-emitting devices by solution processing or vapor phase deposition techniques. Together with the short excitation lifetimes, these properties open up the possibility of constructing inorganic and organic LED devices with very high bandwidth based on phosphors made from linear CAAC coinage metal complexes.

This work was supported by the ERC, the Royal Society, and by the Academy of Finland (Project 251448). M. B. is an ERC Advanced Investigator Award holder (grant no. 338944-GOCAT). The computations were made possible by use of the Finnish Grid Infrastructure resources.
