# Correction: Highly photoluminescent copper carbene complexes based on prompt rather than delayed fluorescence

**DOI:** 10.1039/c8cc90135j

**Published:** 2018-03-28

**Authors:** Alexander S. Romanov, Dawei Di, Le Yang, Julio Fernandez-Cestau, Ciaran R. Becker, Charlotte E. James, Bonan Zhu, Mikko Linnolahti, Dan Credgington, Manfred Bochmann

**Affiliations:** School of Chemistry, University of East Anglia Earlham Road Norwich NR4 7TJ UK m.bochmann@uea.ac.uk; Department of Physics, Cavendish Laboratory, Cambridge University Cambridge CB3 0HE UK; Department of Chemistry, University of Eastern Finland Joensuu Campus FI-80101 Joensuu Finland

## Abstract

Correction for ‘Highly photoluminescent copper carbene complexes based on prompt rather than delayed fluorescence’ by Alexander S. Romanov *et al.*, *Chem. Commun.*, 2016, **52**, 6379–6382.

The original article reported photoluminescence data on cyclic (alkyl)(amino)carbene complexes of copper and gold halides **1a–c** and **2a–c** based on time-correlated single photon counting (TCSPC) measurements carried out over a 10–60 nanosecond timescale which at the time were interpreted as evidence for prompt fluorescence. The authors have remeasured these complexes and this has shown that fluorescence is not the major emission pathway. Given the lifetimes in the microsecond range, the major emission pathway is consistent with phosphorescence. Using either a 370 nm nano-LED or operating in the phosphorimeter mode (with an F1-1029 lifetime emission PMT assembly, using a 450 W Xe lamp with *λ*_ex_ 370 nm, and a decay window of 160–200 μs) gave the lifetimes *τ* as shown in Table 1.

**Table 1:** Excited state lifetimes *τ* [μs] of (CAAC)metal halides

**1a**	**1b**	**1c**	**2a**	**2b**	**2c**
24.9 ± 0.1^a^	22.6 ± 0.1^a^	1.3 ± 0.1 (12%)	2.4 ± 0.1^a^	1.8 ± 0.1 (26%)	2.0 ± 0.1 (47%)
27.2 ± 0.3^b^		17.0 ± 0.1 (88%)^a^		16.7 ± 0.1 (74%)^a^	17.3 ± 0.1 (53%)^a^

a Measured at *λ*_max_ of the emission peak with nano-LED excitation.

b Excitation with a Xe lamp.

The Royal Society of Chemistry apologises for these errors and any consequent inconvenience to authors and readers.

## Supplementary Material

