# Supplementary material for: Highly photoluminescent copper carbene complexes based on prompt rather than delayed fluorescence
Source: Chem Commun (Camb). 2016 Apr 18;52(38):6379–82. doi: 10.1039/c6cc02349e (PMC4920075; doi:10.1039/c6cc02349e)
Supplement: Supplementary file 1 [file CC-052-C6CC02349E-s001.pdf]

Electronic Supplementary Material (ESI) for ChemComm.
This journal is © The Royal Society of Chemistry 2016

Highly photoluminescent copper carbene complexes based on prompt rather than delayed fluorescence

Alexander S. Romanov, Dawei Di, Julio Fernandez-Cestau, Ciaran R. Becker, Charlotte E. James, Le Yang, Bonan Zhu, Mikko Linnolahti,* Dan Credgington* and Manfred Bochmann*

Supporting Information

Synthesis and Characterization of Complexes
Table S1: Thermogravimetric analyses
Figures S1 – S3: UV and photoluminescence spectra
Figures S4, S5: MO representations and excited state structures
Table S2. Structural parameters of the optimized ground and excited state geometries of copper and gold halides
Figure S6: Photoluminescence spectra of 1a at 4 K to 300 K for 420-700 nm.
Figure S7: PL decay lifetimes of 1a and 2a at different temperatures.
Figure S8: Solvent effects on PL
Table S3. Photophysical properties of $(^{Ad}L)MX$ complexes in aerated CH_2Cl_2 solution
Table S4. Binding energies and M–O distances of the S_0 and T_1 states of the THF-coordinated halide complexes
Figure S9. Optimized calculated geometries of the S_0 and T_1 states of THF-coordinated halide complexes.
Table S5. Electrochemical data
Cryogenic and time-resolved PL measurements.
X-ray Crystallography
Figure S10. Crystal structure of independent molecules A for $(^{Ad}L)CuX$ (X = Cl, Br, I).
Table S6: Crystal and refinement data
Table S7. Intermolecular C–H···Hal hydrogen bonds.
Computational details

Synthesis of Complexes

General Considerations. Unless stated otherwise all reactions were carried out in air. Solvents were distilled and dried as required. Sodium *tert*-butoxide and carbazole were purchased and used as received. ^{Ad}CAAC[S1] and (^{Ad}L)AuX (X = Cl, Br, I)[S2] were obtained according to a literature procedures. ^{1}H, ^{13}C{^{1}H} and ^{19}F NMR spectra were recorded using a Bruker Avance DPX-300 MHz NMR spectrometer. ^{1}H NMR spectra (300.13 MHz) and ^{13}C{^{1}H} (75.47 MHz) were referenced to CD_2Cl_2 at δ 5.32 (^{13}C, δ 53.84), C_6D_6 at δ 7.16 (^{13}C, δ 128.4), $CDCl_3$ at δ 7.26 (δ ^{13}C 77.16), or C_6D_5Br at δ 7.30 for the most downfield signal (^{13}C, δ 122.5) ppm. UV-visible absorption spectra were recorded using a Perkin-Elmer Lambda 35 UV/vis spectrometer. Photoluminescence measurements were recorded on a Perkin Elmer LS55 Fluorescence Spectrometer with a solids mount attachment where appropriate. Time resolved fluorescence data for ^{Ad}CAAC were collected on a TCSPC Fluorolog Horiba Jobin Yvon spectrofluorimeter using Horiba Jobin Yvon DataStation v2.4 software. A NanoLED of 370 nm was used as excitation source. The collected data were analysed using a Horiba Jobin Yvon DAS6 v6.3 software. All electrochemical experiments were performed using an Autolab PGSTAT 302N computer-controlled potentiostat. Cyclic voltammetry (CV) was performed using a three-electrode configuration consisting of either a glassy carbon macrodisk working electrode (GCE) (diameter of 3 mm; BASi, Indiana, USA) combined with a Pt wire counter electrode (99.99 %; GoodFellow, Cambridge, UK) and an Ag wire pseudoreference electrode (99.99 %; GoodFellow, Cambridge, UK). The GCE was polished between experiments using alumina slurry (0.3 μm), rinsed in distilled water and subjected to brief ultrasonication to remove any adhered alumina microparticles. The metal electrodes were then dried in an oven at 100 °C to remove any residual traces of water, the GCE was left to air dry and residual traces of water were removed under vacuum. The Ag wire pseudoreference electrodes were calibrated to the ferrocene/ferrocenium couple in MeCN at the end of each run to allow for any drift in potential, following IUPAC recommendations.[S3] All electrochemical measurements were performed at ambient temperatures under an inert Ar atmosphere in MeCN containing the complex under study (0.14 mM) and [n-Bu_4N][PF_6] as supporting electrolyte (0.13 mM). Data were recorded with Autolab NOVA software (v. 1.11). Elemental analyses were performed by the London Metropolitan University. Thermogravimetric analysis (TGA) was performed using a METTLER-TOLEDO TGA-1. The solid sample (approx. 7 mg) was placed in a 70 µL platinum pan and measurements were taken while heating of the sample from 50 °C to 600 °C under nitrogen atmosphere. The background measurement was performed during heating the empty pan over the same temperature range.

Synthesis of (^{Ad}L)CuCl. An oven-dried 100-mL Schlenk flask was equipped with a stirring bar and charged with ^{Ad}L (1.58 g, 4.2 mmol) and CuCl (0.42 g, 4.2 mmol) under an argon atmosphere. Anhydrous THF (20 mL) was added, and the resulting suspension was stirred overnight. The solvent was removed, the residue dissolved in CH_2Cl_2 and filtered through a short pad of silica (1.5 cm). The colorless filtrate was concentrated and hexane was added to precipitate the copper complex, solvents were decanted and the residue dried in vacuum to give a white solid. Yield: 1.86 g (3.9 mmol, 93%).
^{1}H NMR (300 MHz, CD_2Cl_2): δ 7.46 (t, J = 7.2 Hz, 1H, aryl), 7.30 (d, J = 7.2 Hz, 2H, aryl) 3.52 (d, J = 11.4 Hz, 2H, CH_2) 2.81 (sept, J = 6.6 Hz, 2H, C*H*Me_2) 2.23-1.78 (m, 14H, adamantyl CH and CH_2), 1.33 (s, 6H, CMe_2) 1.30 (d, J = 6.6 Hz, 6H, CH*Me*$_2$) 1.29 (d, J = 6.6 Hz, 6H, CH*Me*$_2$). ^{13}C NMR (75 MHz, CD_2Cl_2): δ 253.0 (C carbene), 145.4 (*o*-C), 135.8 (*ipso*-C), 129.9 (*p*-CH), 125.1 (*m*-CH), 78.9 (C_q), 64.9 (C_q), 48.1 (CH_2), 38.9 (CH_2), 37.5 (CH), 36.2 (CH_2), 34.5 (CH_2), 29.54 (CH), 29.40, 28.4, 27.6, 27.0, 22.6 (CH_3). Anal. Calcd. for $C_{27}H_{39}NCuCl$ (476.60): C, 68.04; H, 8.25; N, 2.94. Found: C, 67.91; H, 8.35; N, 3.07.

Synthesis of (^{Ad}L)CuBr. Prepared as described for (^{Ad}L)CuCl from 217 mg (0.58 mmol) of ^{Ad}L and 82.5 mg (0.58 mmol) of CuBr as a white solid. Yield: 0.193 g (0.37 mmol, 64%).
^{1}H NMR (300 MHz, $CDCl_3$) δ 7.40 (t, J = 7.2 Hz, 1H, aryl), 7.25 (d, J = 7.2 Hz, 2H, aryl), 3.57 (d, J = 12.6 Hz, 2H, CH_2), 2.79 (sept, J = 6.6 Hz, 2H, C*H*Me_2), 2.22–1.77 (m, 14H, adamantyl CH, CH_2), 1.34 (s, 6H, CH*Me*$_2$), 1.32 (d, J = 6.6 Hz, 6H, CH*Me*$_2$), 1.29 (d, J = 6.6 Hz, 6H, CH*Me*$_2$). ^{13}C NMR (75 MHz, $CDCl_3$): δ 253.3 (C carbene), 144.9 (*o*-C), 135.2 (*ipso*-C), 129.8 (*p*-CH), 124.9 (*m*-CH), 78.5 (C_q), 64.8 (C_q), 48.1 (CH_2), 38.7 (CH_2), 37.3 (CH), 36.0 (CH_2), 34.4 (CH_2), 29.5 (CH), 29.2, 27.8, 27.23, 27.20, 22.7 (CH_3). Anal. Calcd. for $C_{27}H_{39}NCuBr$ (521.05): C, 62.24; H, 7.54; N, 2.69. Found: C, 62.15; H, 7.68; N, 2.75.

Synthesis of (^{Ad}L)CuI. Prepared as described for (^{Ad}L)CuCl from 199 mg (0.53 mmol) of ^{Ad}L and 100 mg (0.53 mmol) of CuI as a white solid. Yield: 0.170 g (0.30 mmol, 56%).
^{1}H NMR (300 MHz, $CDCl_3$) δ 7.41 (t, J = 7.5 Hz, 1H, aryl), 7.26 (d, J = 7.5 Hz, 2H, aryl), 3.59 (d, J = 12.6 Hz, 2H, CH_2), 2.79 (sept, J = 6.6 Hz, 2H, C*H*Me_2), 2.23 – 1.79 (m, 14H, adamantyl CH, CH_2), 1.36 (s, 6H, CMe_2), 1.35 (d, J = 6.6 Hz, 6H, CH*Me*$_2$), 1.29 (d, J = 6.6 Hz, 6H, CH*Me*$_2$). ^{13}C NMR (75 MHz, $CDCl_3$): δ 254.1 (C carbene), 145.0 (*o*-C), 135.2 (*ipso*-C), 129.8 (*p*-CH), 124.9 (*m*-CH), 78.6 (C_q), 65.0 (C_q), 48.2 (CH_2), 38.8 (CH_2), 37.4 (CH), 36.0 (CH_2), 34.4 (CH_2), 29.5 (CH), 29.2, 27.9, 27.4, 27.2, 22.9 (CH_3). Anal. Calcd. for $C_{27}H_{39}NCuI$ (568.05): C, 57.09; H, 6.92; N, 2.47. Found: C, 57.50; H, 7.09; N, 2.68.

Table S1. Decomposition temperature (T_d, °C) and melting point (T_m, °C) for complexes **1/2 a-f** obtained by thermal gravimetric analysis (TGA) and differential scanning calorimetry (DSC).

	T_d/T_m		T_d/T_m
**1a**	328/280	**2a**	345/305
**1b**	319/276	**2b**	323/300
**1c**	313/273	**2c**	309/303

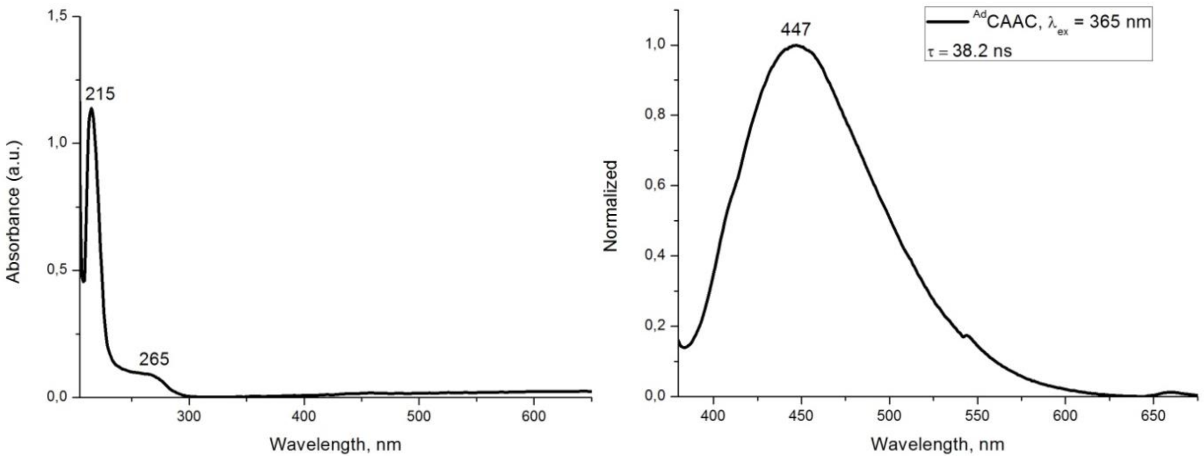

Figure S1. Left: UV-vis spectrum of ^{Ad}CAAC in THF solution. Right: Emission spectrum of ^{Ad}CAAC in the solid state (excitation at λ_{ex} = 365 nm).

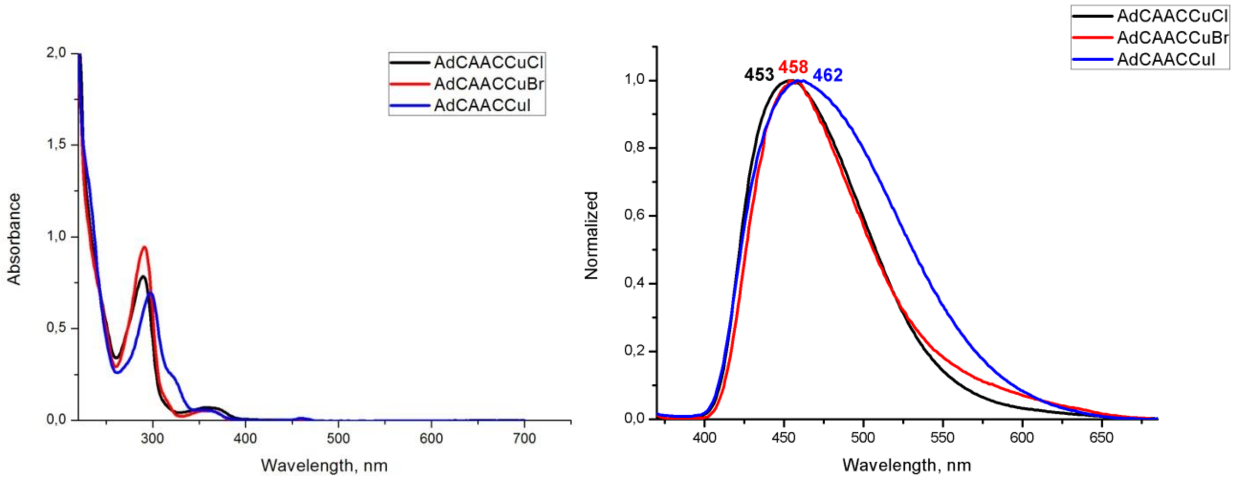

Figure S2. Left: UV-vis spectra of THF solutions of (^{Ad}L)CuX (X = Cl, Br, I). Right: Emission spectra of (^{Ad}L)CuX (X = Cl, Br, I) in the solid state (excited at λ_{ex} = 365 nm).

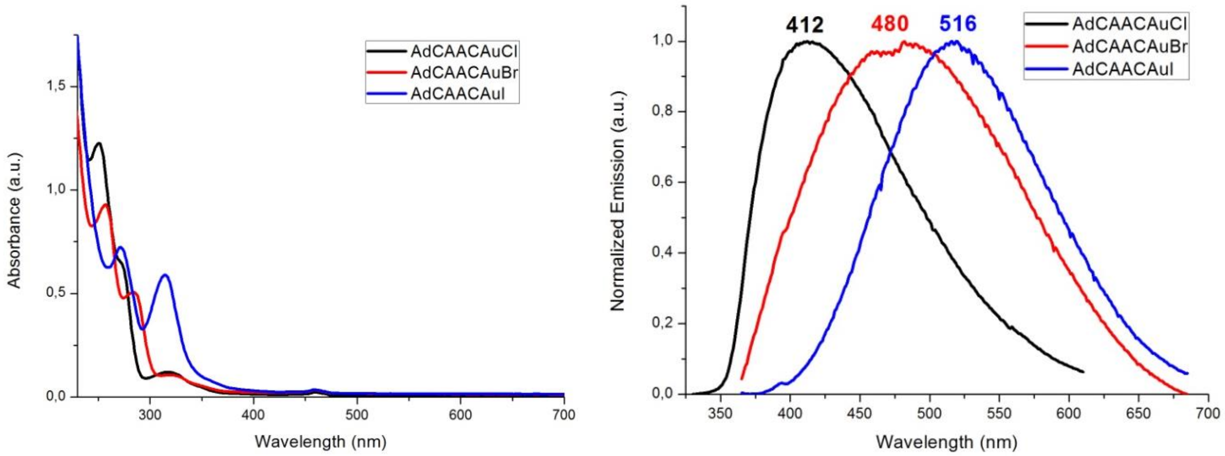

Figure S3. Left: UV-vis spectra of THF solutions of (^{Ad}L)AuX (X = Cl, Br, I). Right: Emission spectra of (^{Ad}L)AuX (X = Cl, Br, I) in the solid state (excited at λ_{ex} = 365 nm).

	HOMO	LUMO
**1a**	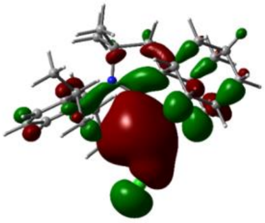	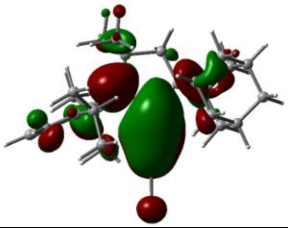
**1b**	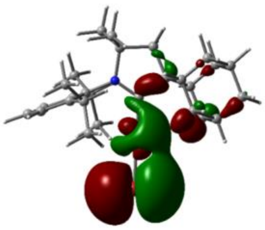	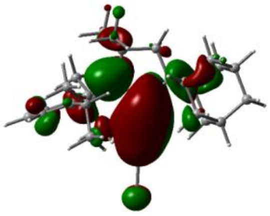
**1c**	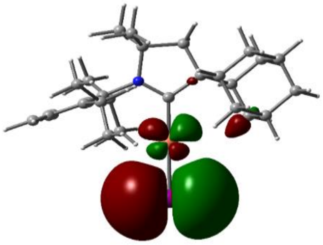	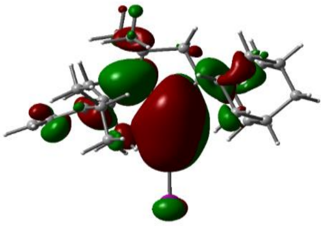
**2a**	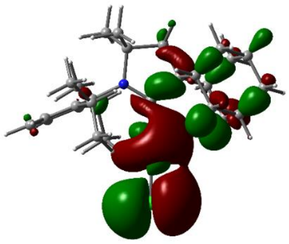	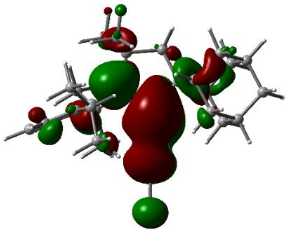
**2b**	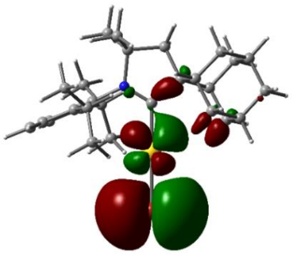	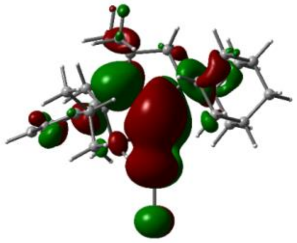
**2c**	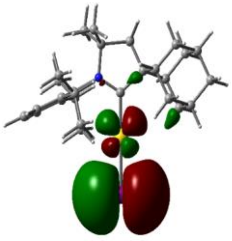	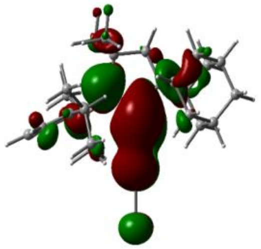

Figure S4. Representations of the HOMOs and LUMOs of the halide complexes (^{Ad}L)MX (M = Cu, Au; X = Cl, Br, I).

Cmpd	S_0	T_1	S_1
**1a**	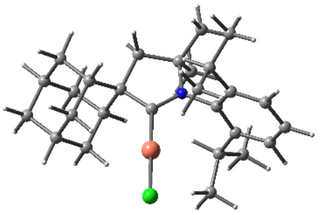	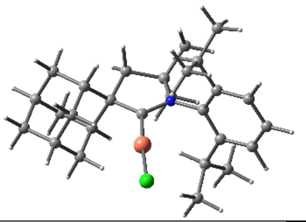	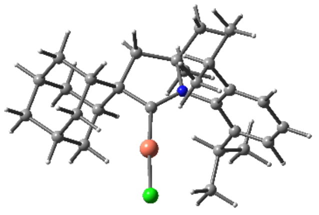
**1b**	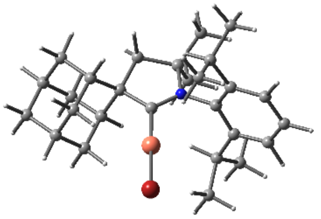	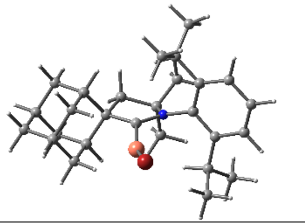	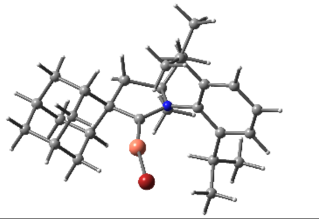
**1c**	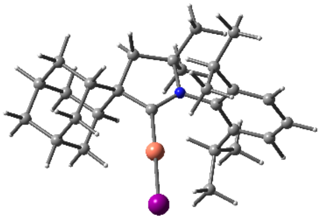	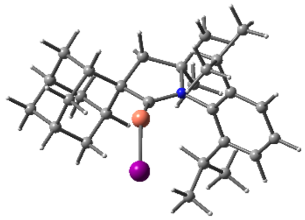	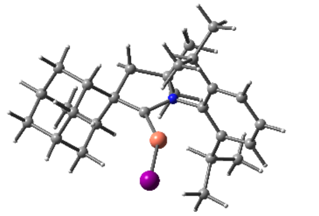
**2a**	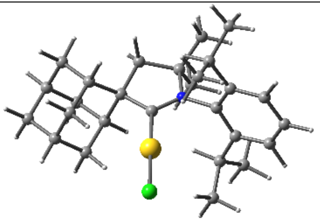	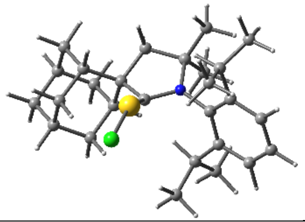	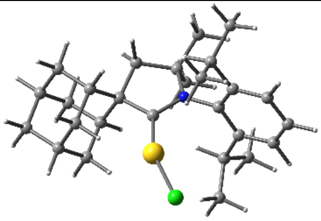
**2b**	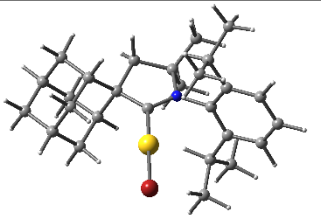	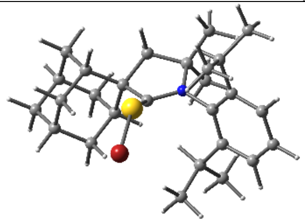	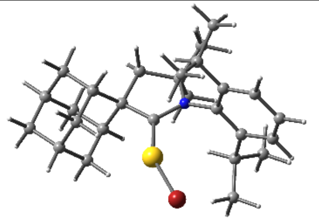
**2c**	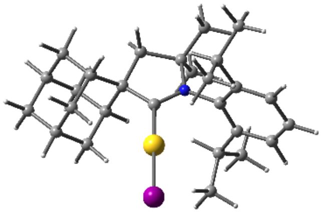	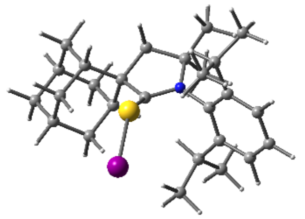	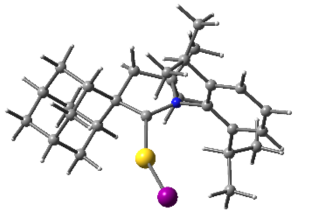

Figure S5. Optimized calculated geometries of the S_0, S_1 and T_1 states of the halide complexes (^{Ad}L)MX (M = Cu, Au; X = Cl, Br, I).

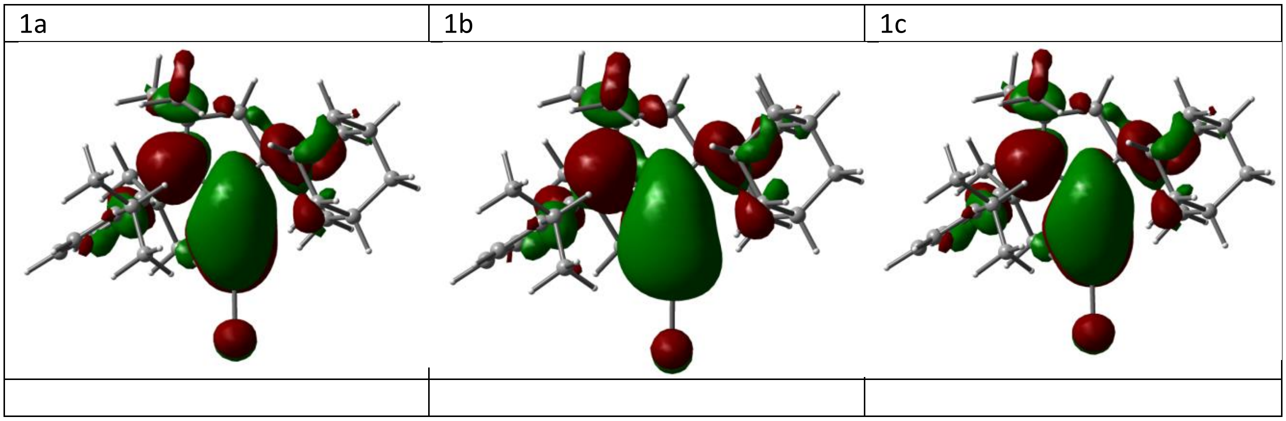

Figure S6. Highest singly-occupied molecular orbitals of **1a - 1c**.

Table S2. Selected structural parameters of the optimized ground and excited state geometries of copper and gold halides

	M–C			M–X			C-M-X	C–M–X calc.			C6–N–C–M		
	S_0	T_1	S_1	S_0	T_1	S_1	Exper.[°]	S_0	T_1	S_1	S_0	T_1	S_1
**1a**	1.894	1.851	1.890	2.111	2.141	2.129	175.33(5)	179.7	175.8	176.2	0.6	4.3	3.0
**1b**	1.902	1.863	1.890	2.248	2.267	2.264	177.59(11)	179.6	156.0	168.0	1.1	31.0	12.8
**1c**	1.913	1.873	1.914	2.426	2.442	2.439	178.52(5)	179.2	144.5	154.2	0.6	28.5	4.0
**2a**	1.989	1.998	1.997	2.277	2.288	2.288		179.7	153.5	159.2	0.5	13.1	7.9
**2b**	1.998	2.004	1.998	2.405	2.418	2.411	178.60(10)	179.8	145.2	156.2	1.5	48.9	9.1
**2c**	2.014	2.013	2.013	2.571	2.586	2.583	179.15(9)	179.8	141.1	153.4	1.6	46.2	33.2

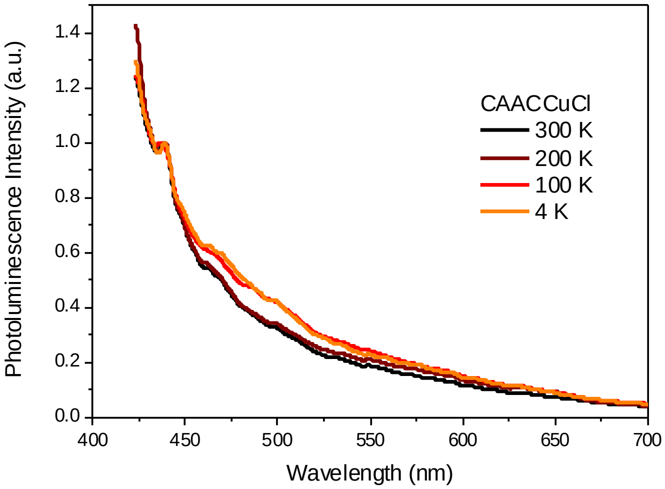

Figure S7. Photoluminescence spectra of **1a** at 4 K to 300 K for the 420-700 nm emission edge. No significant shift is observed, indicating a minimal contribution from phosphorescence.

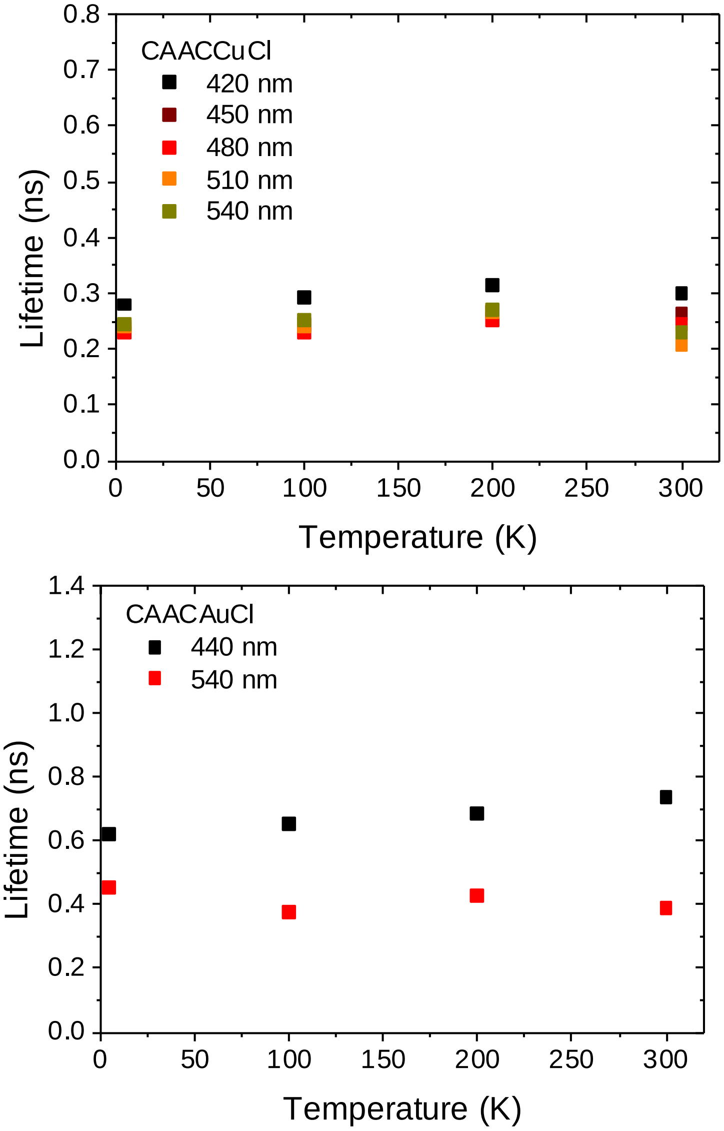

Figure S8. Photoluminescence decay lifetimes of **1a** and **2a** at various emission wavelengths measured using time-correlated single photon counting (TCSPC) as a function of temperature. All decays are temperature-independent within error.

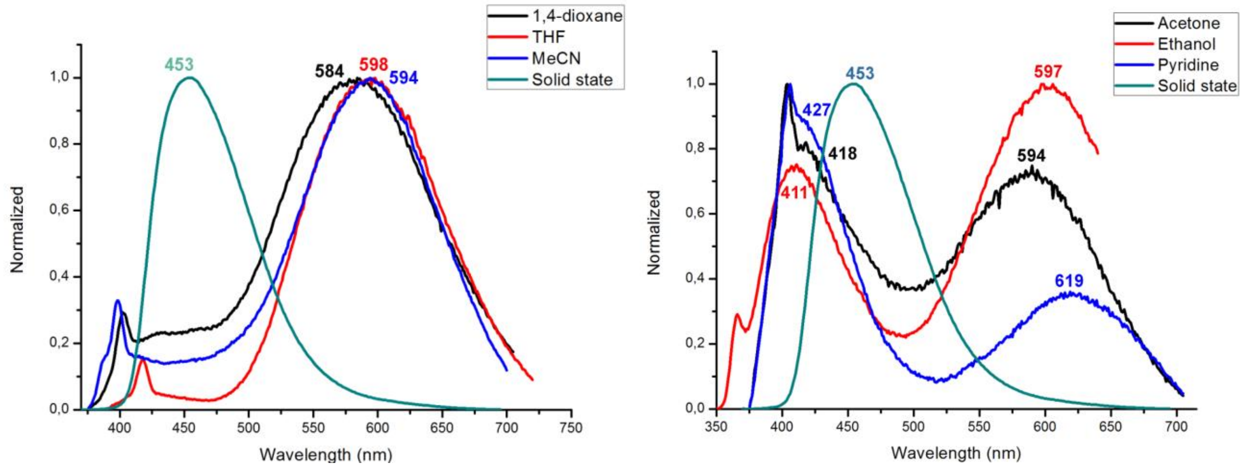

Figure S9. Comparison of emission spectra for ([Ad]L)CuCl in solid state (λ_{ex} = 365 nm) and in various solvents (the small peaks near 400 nm are due to Raman scattering).

Table S3. Photophysical properties of ([Ad]L)MX complexes in aerated CH_2Cl_2 solution.

Complex	λ_{em} (λ_{exc}), nm	Lifetime (ns)[a]
**1a**	456 (290–390)	6 (3%), 160 (97%)
**1b**	453 (300–375)	10 (28%), 72 (72%)
**1c**	445 (290–380)	38 (72%), 146 (28%)
**2a**	480 (300–375)	5 (53%), 25 (47%)
**2b**	513 (280–380)	4 (23%), 33 (77%)
**2c**	544 (290–360)	7 (3%), 175 (97%)

[a] Lifetimes are measured at maximum of λ_{em} and are limited by the instrument response (2 ns)

Table S4. Binding energies and M–O distances of the S_0 and T_1 states of the THF-coordinated halide complexes (^{Ad}L)MX (M = Cu, Au; X = Cl, Br, I).

	Binding energy (kJ mol^{-1})	M–O (Å)
**1a** (T_1)	–46.5	2.174
**1b** (T_1)	–45.7	2.164
**1c** (S_0)	–9.2	2.387
**1c** (T_1)	–43.6	2.176
**2a** (T_1)	–21.3	2.382
**2b** (T_1)	–20.8	2.486
**2c** (T_1)	–20.5	2.443

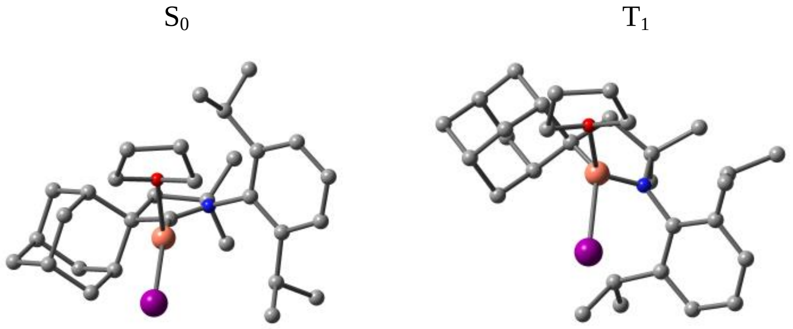

Figure S10. Optimized calculated geometries of the S_0 (Cu-O 2.387) and T_1 (Cu-O 2.176) states of the THF-coordinated halide complex (^{Ad}L)CuI (Cu-O distances: S_0: 2.387 Å; T_1: 2.176 Å). Hydrogens are omitted for clarity.

Table S5. Formal electrode potentials ($E_{1/2}$ for irreversible and for partially reversible processes (*), *V*, *vs*. $FeCp_2$), onset potentials (*E*, *V*, *vs*. $FeCp_2$), E_{HOMO}/E_{LUMO} (*e*V) and band gap values (ΔE, *e*V) for the redox changes exhibited by **1** and **2** in MeCN solution.[a]

Complex	Reduction		E_{LUMO} eV	Oxidation			E_{HOMO} eV	ΔE eV
	$E_{M(I)/M(0)}$	$E_{onset\ red}$		E_{1st}	$E_{onset\ ox}$	E_{2nd}		
**1a**	–2.65	–2.60	–2.79	+0.67	+0.59	–	–5.98	3.19
**1b**	–2.66	–2.61	–2.78	+0.58	+0.49	–	–5.88	3.10
**1c**	–2.56	–2.45	–2.94	+0.17	+0.11	+0.76*	–5.50	2.56
**2a**	–2.57	–2.52	–2.87	+1.46	+1.34	–	–6.73	3.86

**2b**	–2.67*	–2.57	–2.82	+1.43	+1.37	–	–6.76	3.94
**2c**	–2.59*	–2.50	–2.89	+0.86	+0.79	–	–6.18	3.29

[a] Recorded using a glassy carbon electrode, concentration 0.14 m*M*, supporting electrolyte [n-Bu_4N][PF_6] (0.13 *M*), measured at 0.1 V s^{-1}.

Cryogenic and time-resolved PL measurements.

For PL measurements, the samples (spin-cast films on quartz substrates) were photoexcited using a 407 nm pulsed laser with pulse width <200 ps. The time-integrated PL spectra were measured with a spectrograph (SpectraPro 2500i, Princeton Instruments) coupled with a thermo-electronically cooled CCD camera (PIXIS 100-F, Princeton Instruments). A time-correlated single photon counting (TCSPC) setup (Lifespec-ps, Edinburgh Instruments) was used to measure the PL kinetics. The cooling of the sample was provided by a liquid helium cryostat.

X-Ray Crystallography.

Crystals suitable for X-ray study for copper complexes were obtained by layering of CH_2Cl_2 solutions with hexanes, while gold complexes were crystallized by slow evaporation of benzene solutions. Crystals were mounted in oil on glass fibers and fixed on the diffractometer in a cold nitrogen stream. Data were collected on an Oxford Diffraction Xcalibur-3/Sapphire3-CCD diffractometer, using graphite monochromated Mo K_α radiation (λ = 0.71073 Å) at 140 K. Data were processed using the CrystAlisPro-CCD and –RED software.[S4] The principal crystallographic data and refinement parameters are listed in Table S1. The complexes (^{Ad}L)CuX (X = Cl, Br, and I) crystallized with two independent molecules in the unit cell. The structures were solved by direct methods and refined by the full-matrix least-squares against F^2 in an anisotropic (for non-hydrogen atoms) approximation. All hydrogen atom positions were refined in isotropic approximation in "riding" model with the U_{iso}(H) parameters equal to 1.2 $U_{eq}(C_i)$, for methyl groups equal to 1.5 $U_{eq}(C_{ii})$, where $U(C_i)$ and $U(C_{ii})$ are respectively the equivalent thermal parameters of the carbon atoms to which the corresponding H atoms are bonded. All calculations were performed using the SHELXTL software.[S5]

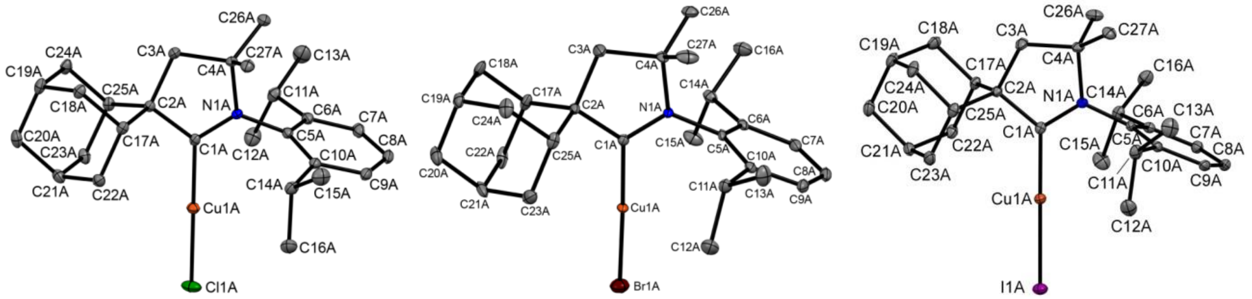

Figure S11. Crystal structure of independent molecules A for ([Ad]L)CuCl / ([Ad]L)CuBr / ([Ad]L)CuI. Ellipsoids are shown at the 50% level. Hydrogen atoms are omitted for clarity. Selected bond lengths [Å] and angles [°]: Cu1A–C1A 1.883(2) / 1.893(4) / 1.9059(17), Cu1A–Hal1A 2.1099(5) / 2.2176(6) / 2.4224(2), C1A–C2A 1.530(2) / 1.523(5) / 1.525(2), C1A–N1A 1.305(2) / 1.301(5) / 1.305(2), C1A–Cu1A–Hal1A 175.33(5) / 177.59(11) / 178.52(5)

Table S6. Summary of crystallographic data and structure refinement.

	([Ad]L)CuCl	([Ad]L)CuBr	([Ad]L)CuI
Empirical formula	$C_{27}H_{39}ClCuN$	$C_{27}H_{39}BrCuN$	$2C_{27}H_{39}CuIN{\cdot}CH_2Cl_2$
Molecular weight	476.58	521.04	1220.99
Crystal system	Monoclinic	Monoclinic	Triclinic
Space group	*P2₁/c*	*P2₁/c*	*P-1*
Crystal colour, habit	colorless/block	colorless/block	colorless/block
Crystal size (mm)	0.48×0.45×0.29	0.48×0.39×0.36	0.33×0.28×0.13
a (Å)	15.9209(1)	16.0878(2)	11.1918(2)
b (Å)	19.4144(2)	19.4137(3)	11.9685(2)
c (Å)	16.2447(2)	16.2912(3)	20.7901(4)
α (°)	90	90	101.520(2)
β (°)	103.631(1)	103.540(2)	94.798(1)
γ (°)	90	90	94.419(1)
V (Å^3)	4879.72(8)	4946.71(13)	2706.59(8)
Z	8	8	2
D_{calc} (g cm^{-3})	1.297	1.399	1.498
$2\theta_{max}$ (°)	28.00	27.00	28.00
Abs. coeff., μ(Mo-K_α) (cm^{-1})	1.018	2.512	2.061
T_{max}/T_{min}	0.7566/0.6406	0.4649/0.3785	0.7754/0.5495
Number of collected	80046	71950	47637

Number of independent	11765	10788	13045
Number of observed	9821	8881	11265
R_{int}	0.0411	0.0411	0.0227
Number of parameters	553	553	580
R_1 (on F for observed	0.0334	0.0512	0.0222
wR_2 (on F^2 for all reflexions)	0.0838	0.1574	0.0574
Weighting scheme			
A	0.04	0.08	0.03
B	3.0	21.4	0.7
F(000)	2032	2176	1244
Goodness-of-fit	0.994	1.034	1.004
$\Delta\rho_{max}/\Delta\rho_{max}$ (e Å^{-3})	0.820/-0.272	1.278/-1.861	0.826/-0.624

Table S7. Intermolecular C–H···Hal (Hal = Cl, Br, I) hydrogen bonds (Å, °) found for **1/2 a-c**.

Compound	D–H···A	d(D–H)	d(H···A)	Δ*	d(D···A)	DHA [°]
**1a**	C17B–H17B^{a}···Cl1A	1.00	2.91	–0.04	3.596(2)	128
**1b**	C8A–H8A···Br1A^{b}	1.00	3.03	–0.02	3.034(3)	148
	C17B–H17B^{c}···Br1B	1.00	2.94	–0.11	3.627(5)	128
**1c**·CH_2Cl_2	C9A–H9A^{d}···I1B	1.00	3.06	–0.12	3.850(2)	144
**2a**	C22A–H24A^{e}···Cl1**	1.00	2.90	–0.03	3.592(4)	128
**2b**	C9A–H9A^{f}···Br1B	1.00	2.98	–0.07	3.760(2)	142
**2c**	none	–	–	–	–	–

* Difference between spherical van der Waals radii of H atom and halides proposed by Bondi (A. Bondi, *J. Phys. Chem.*, 1964, **68**, 441–451; R.S. Rowland, R. Taylor, *J. Phys. Chem.*, 1996, **100**, 7384–7391)

** G.D. Frey, R.D. Dewhurst, S. Kousar, B. Donnadieu, G. Bertrand, *J. Organomet. Chem.*, 2008, **693**, 1674.

Symmetry transformations used to generate equivalent atoms: ^{a} = –1 + x, y, 1 + z; ^{b} = 2 – x, 1 – y, 2 – z; ^{c} = 1 – x,1 – y, 2 – z; ^{d} = x,1 + y, z; ^{e} = x, 3/2 – y, 1/2 + z; ^{f} = x, –1 + y, z;

Computational details

The ground states were fully optimized by the hybrid density functional PBE0[S6] method in combination with def2-TZVP basis set of Ahlrichs and coworkers.[S7] Relativistic effective core potentials of 28 and 60 electrons were used to describe the core electrons of I and Au, respectively.[S8] The excited states were studied similarly by time-dependent DFT.[S9] The methods and basis sets have been previously employed with success in studies of luminescent Cu- and Au-complexes.[S10] All calculations were carried out by Gaussian 09.[S11]

References:

S1 (a) Lavallo, V.; Canac, Y.; Prasang, C.; Donnadieu, B.; Bertrand, G. Angew. Chem., Int. Ed. 2005, 44, 5705. (b) Jazzar, R.; Dewhurst, R. D.; Bourg, J.-B.; Donnadieu, B.; Canac, Y.; Bertrand, G. Angew. Chem., Int. Ed. 2007, 46, 2899. (c) Jazzar, R.; Bourg, J.-B.; Dewhurst, R. D.; Donnadieu, B.; Bertrand, G. J. Org. Chem. 2007, 72, 3492. (d) Back, O.; Henry-Ellinger, M.; Martin, C. D.; Martin, D.; Bertrand, G. Angew. Chem., Int. Ed. 2013, 52, 2939.

S2 A. S. Romanov, M. Bochmann, *Organometallics* **2015**, *34*, 2439.

S3 G. Gritzner, J. Kůta, Electrochim. Acta 1984, 29, 869.

S4 *Programs CrysAlisPro*, Oxford Diffraction Ltd., Abingdon, UK (2010).

S5 Sheldrick, G.M. SHELX-97 and SHELX-2014 – Programs for crystal structure determination (SHELXS) and refinement (SHELXL), *Acta Cryst.* **2008**, *A64*, 112.

S6 (a) Perdew, J. P.; Burke, K.; Ernzerhof, M. *Phys. Rev. Lett.* **1996**, *77*, 3865. (b) Adamo, C.; Barone, V. *J. Chem. Phys.* **1999**, *110*, 6158.

S7 (a) Weigend, F.; Häser, M.; Patzelt, H.; Ahlrichs, R. *Chem. Phys. Lett.* **1998**, *294*, 143. (b) Weigend, F.; Ahlrichs, R. *Phys. Chem. Chem. Phys* **2005**, *7*, 3297.

S8 (a) Peterson, K. A.; Figgen, D.; Goll, E.; Stoll, H.; Dolg, M. *J. Chem. Phys* **2003**, *119*, 11113. (b) Andrae, D.; Haeussermann, U.; Dolg, M.; Stoll, H.; Preuss, H. *Theor. Chim. Acta* **1990**, *77*, 123.

S9 Furche, F.; Rappoport, D., Density functional methods for excited states: equilibrium structure and electronic spectra. In Computational Photochemistry; Olivuccim M., Ed.; Elsevier: Amsterdam, 2005; pp. 93–128.

S10 (a) Koshevoy, I. O.; Lin, Y.-C.; Karttunen, A. J.; Chou, P.-T.; Vainiotalo, P.; Tunik, S. P.; Haukka, M.; Pakkanen, T. A. *Inorg. Chem.* **2009**, *48*, 2094. (b) Koshevoy, I. O.; Chang, Y.-C.; Chen, Y.-A.; Karttunen, A. J.; Grachova, E. V.; Tunik, S. P.; Jänis, J.; Pakkanen, T. A.; Chou, P.-T. *Organometallics* **2014**, *33*, 2363.

S11 Frisch, M. J.; Trucks, G. W.; Schlegel, H. B.; Scuseria, G. E.; Robb, M. A.; Cheeseman, J. R.; Scalmani, G.; Barone, V.; Mennucci, B.; Petersson, G. A.; Nakatsuji, H.; Caricato, M.; Li, X.; Hratchian, H. P.; Izmaylov, A. F.; Bloino, J.; Zheng, G.; Sonnenberg, J. L.; Hada, M.; Ehara, M.; Toyota, K.; Fukuda, R.; Hasegawa, J.; Ishida, M.; Nakajima, T.; Honda, Y.; Kitao, O.; Nakai, H.; Veven, T.; Montgomery, J. A.; , Jr.; Peralta, J. E.; Ogliaro, F.; Bearpark, M.; Heyd, J. J.; Brothers, E.; Kudin, K. N.; Staroverov, V. N.; Keith, T.; Kobayashi, R.; Normand, J.; Raghavachari, K.; Rendell, A.; Burant, J. C.; Iyengar, S. S.; Tomasi, J.; Cossi, M.; Rega, N.; Millam, J. M.; Klene, M.; Knox, J. E.; Cross, J. B.; Bakken, V.; Adamo, C.; Jaramillo, J.; Gomperts, R.; Stratmann, R. E.; Yazyev, O.; Austin, A. J.; Cammi, R.; Pomelli, C.; Ochterski, J. W.; Martin, R. L.; Morokuma, K.; Zakrzewski, V. G.; Voth, G. A.; Salvador, P.; Dannenberg, J. J.; Dapprich, S.; Daniels, A. D.; Farkas, O.; Foresman, J. B.; Ortiz, J. V.; Cioslowski, J.; Fox, D. J. Gaussian 09, Revision C.01; Gaussian, Inc., Wallingford, CT, 2010.
